# Fluorescein Leakage and Optical Coherence Tomography Angiography Features of Microaneurysms in Diabetic Retinopathy

**DOI:** 10.1155/2022/7723706

**Published:** 2022-01-13

**Authors:** Ruoyu Chen, Anyi Liang, Jie Yao, Zicheng Wang, Yesheng Chen, Xuenan Zhuang, Yunkao Zeng, Liang Zhang, Dan Cao

**Affiliations:** ^1^Department of Ophthalmology, Guangdong Provincial People's Hospital, Guangdong Academy of Medical Sciences, Guangzhou 510080, China; ^2^The Second School of Clinical Medicine, Southern Medical University, Guangzhou 510515, China; ^3^Shantou University Medical College, Shantou 515041, China; ^4^School of Medicine, South China University of Technology, Guangzhou 510080, China; ^5^Guangdong Cardiovascular Institute, Guangzhou 510000, China

## Abstract

**Results:**

Thirty-six, fifty-two, and seventy-nine MAs showed no, mild, and severe leakage on FA, respectively. Most MAs (61.7%) were centered in the inner nuclear layer. Cystoid spaces were observed adjacent to 60 (35.9%) MAs. MAs with severe leakage had a statistically higher flow proportion compared to MAs with no or mild leakage (both *P* < 0.001). Only 112 MAs (67.1%) were visualized in the OCTA en face images, while 165 MAs (98.8%) could be visualized in the OCT en face images. The location of MAs did not associate significantly with FA leakage status. The presence of nearby cystoid spaces and higher flow proportion by OCT B-scan with flow overlay correlated significantly with FA leakage status.

**Conclusion:**

The flow proportion of MAs observed on OCT B-scans with flow overlay might be a potential biomarker to identify leaking MAs. A combination of OCT B-scan, OCT en face, and OCTA en face images increased the detection rate of diabetic MAs in a noninvasive way.

## 1. Introduction

Diabetic retinopathy (DR) is the leading cause of blindness in working age population around the world [1, 2]. MA presents as a small round deep-red dot on fundus color photograph, which is the initial sign appearing in DR patients, as well as the hallmark of clinical diagnosis of DR [3]. Fluorescein angiography (FA) can detect about twice as many MAs as on fundus photograph, which makes it a better tool to discern subtle microvascular abnormalities in DR. Fluorescein leakage from MAs was positively associated with capsular structure disorder and blood-retina barrier breakdown, resulting in vision-threatening focal or diffuse retinal edema [4]. Focal laser therapy guided by FA plays an important role in diabetic macular edema (DME) management, which can accurately close leaking MAs and reduce leakage through the disrupted blood–retinal barrier [5]. However, FA is unsuitable for regular screening of DR because of its potential adverse reactions.

Structural optical coherence tomography (OCT) is a complementary modality that can visualize MAs in a noninvasive way. MAs appear as round or oval well-demarcated intraretinal hyperreflective lesions in OCT B-scan. Optical coherence tomography angiography (OCTA) is another noninvasive imaging technique that provides three-dimensional images of the retinal and choroidal microvasculature. Recently, OCTA has displayed its effectiveness in monitoring DR progression and provided more details of microvascular alterations than FA [6–8]. Now the review software implemented in OCTA enables us to observe individual structural OCT B-scans with flow overlay. AngioAnalytics software built in AngioVue OCTA system, which provides fine histologic visualization of both the retinal microstructure and the blood flow at the same time. However, one of the major disadvantages of OCTA is its inability to visualize the leakage.

To our knowledge, there is no prior study correlating the MAs on OCTA en face images, OCT en face images, OCT B-scan with flow overlay, and FA images. The purpose of this study was to find out whether certain characteristics of MAs on OCTA en face images, and OCT B-scan with flow overlay could reflect the leakage status of MAs on FA images.

## 2. Methods

### 2.1. Subjects

In this retrospective, cross-sectional observational study, thirty-nine patients with type 2 diabetes mellitus (T2DM) and complicated with nonproliferative diabetic retinopathy (NPDR) were included. The diagnosis of T2DM was established according to the diagnostic criteria of American Diabetes Association [9]. Informed consent was obtained from each study participant before examination. Diagnosis and classification of DR were confirmed according to the international clinical diabetic retinopathy and diabetic macular edema disease severity scales [10].

All enrolled subjects were evaluated and underwent successively OCTA and FA imaging at the Ophthalmology Department, Guangdong Provincial People's Hospital, Guangzhou between June 1, 2019 and December 30, 2019. One eye of each patient was randomly selected if both eyes were eligible. The OCTA and FA imaging were evaluated by two masked retina specialists (DC and LZ) independently, and in case of disagreement, there was open adjudication until a consensus was established. The case series were performed according to the Declaration of Helsinki and was approved by the Research Ethics Committee of Guangdong Provincial People's Hospital (registration number: GDREC2018380H (R2)). Eyes with MAs with good-quality FA and OCTA images available were included.

The exclusion criteria were as follows: (1) patients with other retinal vascular diseases; (2) patients with history of intraocular surgeries including antivascular endothelial growth factor (VEGF) treatment, etc.; (3) patients treated with direct photocoagulation of the MAs, or focal/grid photocoagulation, and/or panretinal photocoagulation within 6 months; and (4) patients with ocular conditions that affect imaging of OCTA (scan quality < 6, e.g. advanced cataract).

### 2.2. Image Acquisition

All participants were tested for best corrected visual acuity (BCVA), intraocular pressure (IOP), and refractive error (measured with autorefractometry). Slit-lamp and fundus examinations using direct and/or indirect ophthalmoscope were performed. ETDRS 35 degree 7-standard fields color retinal photographs (Topcon TRC; Topcon, Tokyo, Japan) were obtained from each participant.

Fifty-five-degree FA images were obtained using Heidelberg Retinal Angiography 2 (Spectralis HRA2; Heidelberg Engineering, Heidelberg, Germany). MAs were detected as hyperfluorescent dots in the early phase of FA imaging, and leakage was graded as no, mild, or severe by comparing the FA images of the MAs in the early phase with those in the late phase, as described by Wang and colleagues [11].

OCTA examinations were conducted at the same day after pupil dilation and before the FA acquisition by using AngioVue OCTA system (RTVue-XR Avanti; Optovue, Fremont, CA, USA). We chose the macular HD 6 mm × 6 mm program, which uses an 840 nm light source and provides 70,000 A scans/second. Superficial vascular complex (SVC) and deep vascular complex (DVC) were automatically generated by the built-in software. SVC is defined as a slab extending from internal limiting membrane (ILM) to 10 *μ*m above inner plexiform layer (IPL). DVC is a slab extending from 10 *μ*m above IPL to 10 *μ*m below outer plexiform layer (OPL).

The built-in AngioAnalytics software (version 2017.1.0.151) was adopted which included important advance in three-dimensional projection artifact removal (3D PAR). With 3D PAR, projection artifacts are minimized pixel-by-pixel throughout the entire OCTA volume, facilitating clearer visualization of vascular structures in en face images and in B-scans at all depths. MAs were classified according to three types of shape: nodular type, comma-like type, and absent type. MAs that were visible as nodular findings on OCTA en face images were defined as the nodular type. MAs presented as coil-shaped, comma-shaped, semilunar, crescent, or earlobe-like shape were all defined as the comma-like type. MAs that could not be not be confirmed on OCTA en face images were defined as the absent type [12].

The 6 mm × 6 mm AngioRetina scan could provide 400 OCT B-scans spanning 15 *μ*m apart as well as OCT B-scans with flow overlay. The flow information can be visualized in the cross-sectional B-scans, allowing us to analyze retinal microstructure together with perfused retinal vasculature.

**Figure 1:**
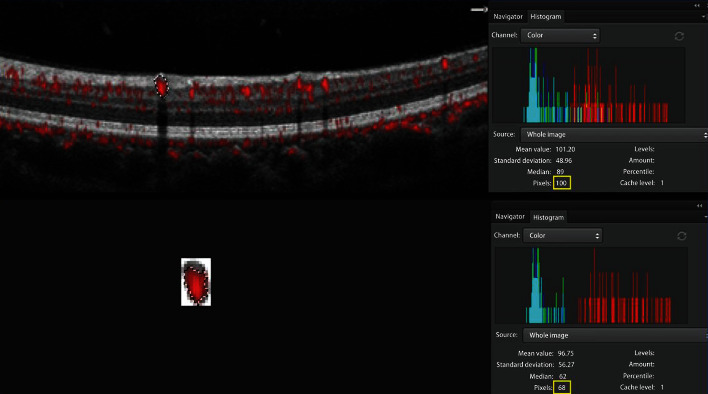
The measurement of flow proportion. MA area and flow area were measured using a Photoshop program. Every MA area in OCT B-scan with flow overlay was dragged using the Photoshop dragging tool and a tablet pen. The histogram of every dragged image was examined and the pixel value of each dragged area was obtained. After that, the selected MA area was cropped out using the Photoshop cropping tool and was pasted to a new image. The flow area in the new MA image was also dragged using the Photoshop dragging tool, and the pixel value of each dragged area was calculated in the histogram panel. Flow proportion was defined as the ratio of flow area divided by MA area. The flow proportion of the MA selected in the figure was 68% (68 pixels/100 pixels).

Flow proportion was defined as the ratio of flow area divided by MA area. MA area and flow area were measured using Photoshop (AdobeSystems, San Jose, CA, USA). Every MA area in the OCT B-scan image with flow overlay was dragged using the Photoshop dragging tool (Figure 1), and a color histogram of each dragged image was examined. The pixel value of each dragged area in the individual frames was calculated and defined as the total area of each MA. After that, the selected MA area was cropped out using the Photoshop cropping tool and was pasted to a new image. The flow area in the new MA image was also dragged using the Photoshop dragging tool, and the pixel value of each dragged area was calculated in the color histogram panel and defined as the area of flow.

**Figure 2:**
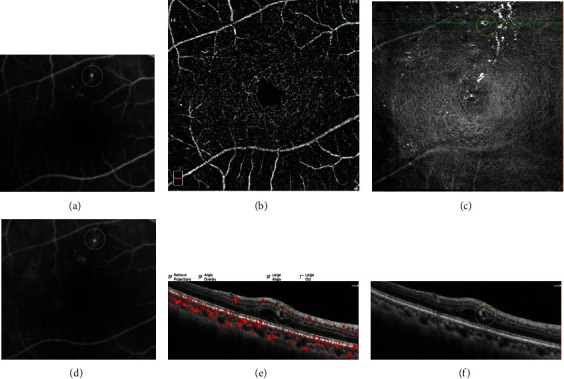
A typical diabetic microaneurysm with no leakage seen by FA (yellow circle (a), early phase of FA (d), late phase of FA). The microaneurysm was not visualized in OCTA en face image (b). On OCT en face image, the microaneurysm appeared as luminescent ring-shaped morphology (c). The microaneurysm was recognized as an oval well-demarcated lesion on OCT B-scan (f), and the lumen was devoid of flow signal on OCT B-scan with flow overlay (e).

**Figure 3:**
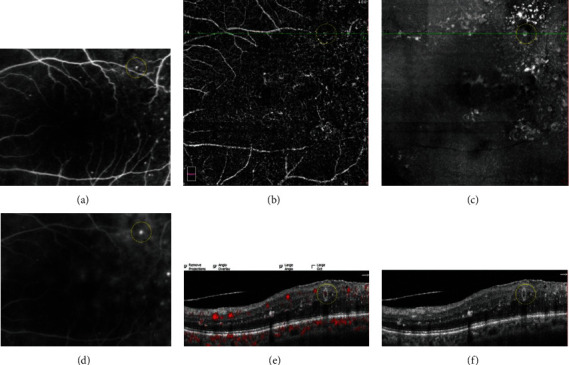
A typical diabetic microaneurysm with mild leakage on FA (yellow circle (a), early phase of FA (d), late phase of FA). The microaneurysm was visualized as comma-like type in OCTA en face image (yellow circle (b)). On OCT en face image, the microaneurysm appeared as a luminescent spot (yellow circle (c)). The microaneurysm was recognized an oval well-demarcated lesion on OCT B-scan (yellow circle (f)), and the flow proportion of the microaneurysm was 24.8% on OCT B-scan with flow overlay (yellow circle (e)).

**Figure 4:**
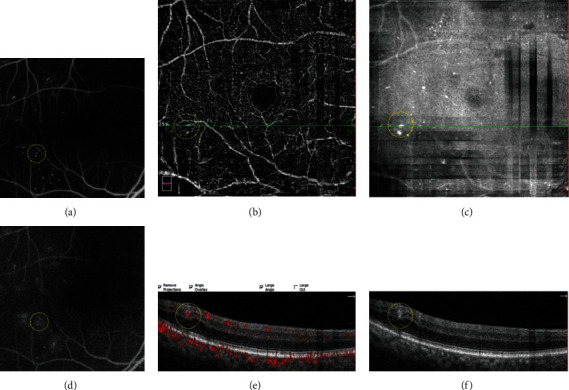
A typical diabetic microaneurysm with severe leakage on FA (yellow circle (a), early phase of FA (d), late phase of FA). The microaneurysm was visualized as nodular type in OCTA en face image (yellow circle (b)). On OCT en face image, the microaneurysm appeared as a luminescent spot (yellow circle (c)). The microaneurysm was recognized a thin-walled oval lesion on OCT B-scan with a retinal cyst nearby (yellow circle (f)), and the flow proportion of the microaneurysm was 57.8% on OCT B-scan with flow overlay (yellow circle (e)).

To compare the MAs seen on FA to those seen on OCT en face/OCTA en face/OCT B-scan, we superimposed the FA macular vascular landmarks onto the vascular landmarks of the OCTA SVC and vascular landmarks on the near infrared images. In addition, we observed cystoid spaces surrounding the MAs on the OCT B-scan images, and we determined whether surrounding cystoid spaces was present or absent. We also analyzed the distributions of MAs by defining which layer the center of each MA went through (Figures 2–4).

### 2.3. Statistical Analysis

Statistical analysis was performed with SPSS software package version 19.0 (SPSS. Inc., Chicago, IL, USA). The Wilcoxon signed rank test was used to compare the morphology of the MAs in the OCTA en face images, FA findings, distribution of MAs (OCT B-scan), flow proportion of MAs, and presence of nearby cystoid spaces around the MAs. For all the tests, *P* < 0.05 was considered statistically significant.

## 3. Results

### 3.1. Baseline Data

A total of 39 eyes from 39 DR patients were included in the study, comprising of 10 eyes with mild NPDR (25.6%), 16 eyes with moderate NPDR (41.1%), and 13 eyes with severe NPDR (33.3%). Gender distribution, mean age, and BCVA of patients were listed in Table 1. The mean age, the mean duration of T2DM, and the mean glycosylated hemoglobin (HbA1c) of enrolled subjects was 58.3 ± 7.2 years, 8.6 ± 4.3 years, and 8.9 ± 2.6%, respectively.

### 3.2. Fluorescein Angiography

A total of 167 MAs in 39 diabetic eyes were evaluated by FA and OCTA imaging simultaneously. Seventy-nine out of 167 MAs showed severe leakage (47.4%), followed by 52 MAs with mild leakage (31.1%). The number of MAs with no leakage on FA was 36 (21.5%).

### 3.3. Distribution of Diabetic MAs

**Figure 5:**
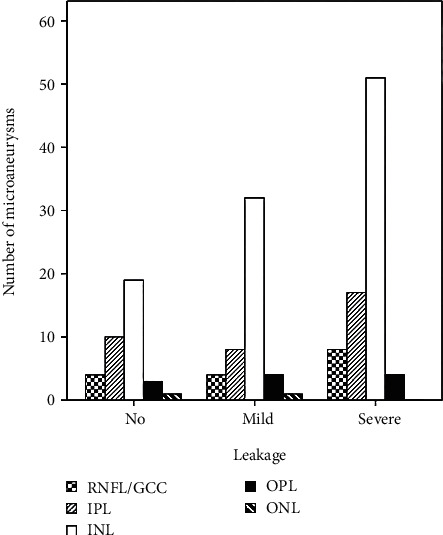
Distribution of the center of diabetic microaneurysms (*N* = 167) by retinal layers. RNFL: retinal nerve fiber layer; GCC: ganglion cell layer; IPL: inner plexiform layer; INL: inner nuclear layer; OPL: outer plexiform layer; ONL: outer nuclear layer.

The center of diabetic MAs was observed mostly in inner nuclear layer (103/167, 61.7%), followed by inner plexiform layer (35/167, 20.9%), retinal nerve fiber layer/ganglion cell layer (16/167, 9.6%), outer plexiform layer (11/167, 6.6%), and outer nuclear layer (2/167, 1.2%) (Figure 5). The distribution of MAs was not significantly correlated with FA leakage status (*P* = 0.774).

### 3.4. Adjacent Cystoid Spaces of MAs by OCT B-Scan

**Figure 6:**
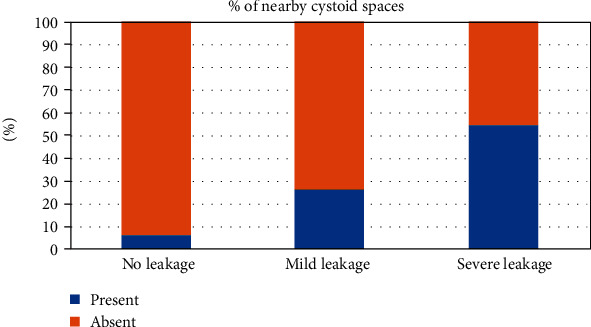
Percentages of presence of nearby cystoid spaces around diabetic microaneurysms in different fluorescein leakage groups.

Cystoid spaces were observed adjacent to 60 MAs (35.9%). Adjacent cystoid spaces on OCT B-scan were associated with increasing FA leakage status (*P* < 0.001). In the severe leakage group, 55.7% MAs had nearby cystoid spaces, while only 5.6% MAs with no leakage had nearby cysts (Figure 6).

### 3.5. Flow Proportion of Diabetic MAs

**Figure 7:**
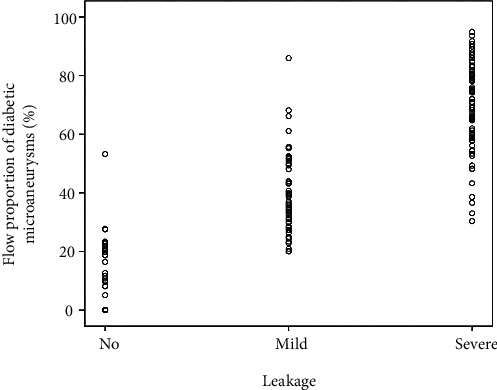
Scatter plot of flow proportion of diabetic microaneurysms in different fluorescein leakage groups.

MA area and flow area of each MA were measured using Photoshop, and the flow proportion was recorded. MAs with severe leakage had a significantly higher flow proportion compared to MAs with no or mild leakage (both *P* < 0.001) (Table 2, Figure 7).

### 3.6. Visualization of Diabetic MAs Using Different Imaging Modalities

Of the 167 diabetic MAs, only 112 MAs (67.1%) were visualized in the OCTA en face images, while 165 MAs (98.8%) could be visualized in the OCT en face images. OCT en face images had a 100% detection rate of MAs with no leakage and mild leakage in FA. However, only 22.2% of MAs with no leakage and 67.3% of MAs with mild leakage could be seen in the OCTA en face images. With regard to the MAs with severe leakage, OCTA en face images could detect 87.3% of them and OCT en face images could detect 97.5% of them (Table 3).

### 3.7. Type of MA Visualized by OCTA En Face Image

**Figure 8:**
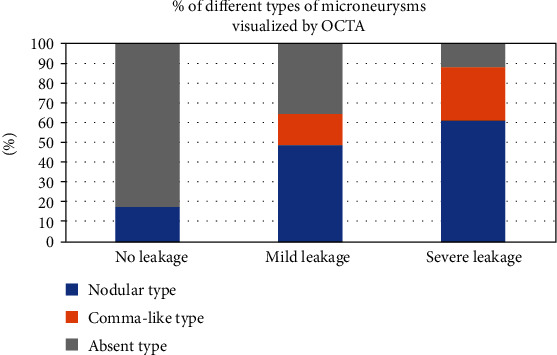
Percentages of different types of microaneurysms visualized by OCTA in different fluorescein leakage groups. OCTA: optical coherence tomography angiography.

Altogether there were 81 nodular type (48.5%), 31 comma-like type (18.6%), and 55 absent type (32.9%) MAs in the 167 angiographically visible MAs. The type of MAs visualized by OCTA en face images was significantly correlated with FA leakage status (*P* < 0.001). MAs with no leakage showed more absent type on OCTA en face than MAs with mild or severe leakage (Figure 8).

## 4. Discussion

This study outlined particular characteristics of diabetic MAs on OCTA en face and OCT B-scan with flow overlay, with the aim of correlating these features of MAs on noninvasive examinations to the leakage status of MAs on FA images. Our results demonstrated that only 67.1% MAs visualized on FA were detected by OCTA, while 98.8% MAs were seen in OCT en face images. MAs with severe leakage are easier to be recognized in OCTA en face images compared to those with no leakage (87.3% versus 22.2%). Besides, most MAs centered in DVC (INL>IPL>RNFL/GCC>OPL) and the location of MAs did not seem to affect their leakage status. However, increasing FA leakage of MAs was associated with adjacent cystoid spaces and higher flow proportion in the OCT B-scan with flow overlay.

Diabetes is characterized by vessel basement membrane thickening and selective degeneration with pericyte loss, which leads to asymmetric dilatations of the capillary wall where it is weakened. The loss of supporting pericytes and localized increase in hydrostatic pressure subsequently caused the formation of MAs. Histological studies showed that the MAs ranged in morphology from thin-walled, cellular forms to dense, acellular, hyalinized forms. Lumen contents of microaneurysms may be comprised of polymorphonuclear cells, red blood cells, fibrotic cells, thrombi, or lipid aggregates. And most of MAs were observed in the inner layers of the retina although, less frequently, they could be found in the outer plexiform layer [13–15].

Our results demonstrated that adjacent cystoid spaces were associated with increased leakage of MAs on FA images, which is in accordance with previous studies that the leakage of MAs is the contributing factor to extracellular fluids aggregation and diabetic macular edema [16]. Thus, monitoring the extent of leakage of MAs may be advantageous to macular edema treatment. In the clinical setting, FA can provide good capillary images showing leakage of MAs. Focal laser photocoagulation targeting leaking diabetic MAs with FA guidance can increase accuracy and hit rate of laser. Moreover, FA can be used to evaluate the closure of MAs and monitor the progression of DME [5]. However, FFA is invasive, time-consuming, and with the potential side effect that is not suitable in certain conditions, such as pregnancy and kidney failure. Thus, it could not be applied to every patient in daily practices.

Ito et al. [17] evaluated the characteristics of diabetic MAs seen on color fundus photography, FA, and spectral domain OCT (SD-OCT). They found that focal fluorescein leakage from MAs was associated positively with the absence of a capsular structure, hyperreflective spots in the lumen, and nearby cystoid spaces. Horii et al. [18] characterized MAs in DR depicted by SD-OCT and categorized the MAs into 3 types (complete ring sign, incomplete ring sign, and no structure) according to the status of the capsule structure, and they showed that MAs with the ring sign were positively correlated with nearby cystoid spaces and protrusion into the cystoid spaces.

A previous study had assessed the internal reflectivity of MAs on SD-OCT B-scan and compared them with MA visualization on the OCTA images. The authors found that MAs with internal hyporeflectivity on SD-OCT B-scan had a significantly lower detection rate on OCTA (66.7%) compared to MAs with internal hyperreflectivity (88.9%) or moderate reflectivity (88.9%) [19]. They further investigated the progression of diabetic MAs according to the SD-OCT and OCTA characteristics and found that the presence of flow, the visibility, and the deep location were strongly associated with the development of extracellular fluid at 12 months [20]. However, the internal reflectivity of MAs on OCT B-scan is not a true reflection of blood flow proportion of MAs.

Recently, OCTA has become more available and popular. Because of its rapidness and noninvasiveness, OCTA can provide much of the information given by FA. However, OCTA cannot visualize the leakage. Now, the OCTA with AngioVue provides 3D PAR, facilitating clearer visualization of vascular structures in en face images and in B-scans at all depth. The OCT B-scan with flow overlay generated by AngioVue device offers a new aspect for us to investigate the flow signal and microstructure of MAs at the same time. Our study defined flow proportion as the ratio of flow area divided by MA area in OCT B-scan images with the help of Photoshop, and the results indicated that high flow proportion of MAs on segmentation OCT B-scan with flow overlay correlated well with increasing leakage of MAs on FA. Therefore, OCT B-scan with flow overlay could be a clinically useful tool to predict the leakage status of diabetic MAs and to monitor the turnover of MAs. If we combine the flow proportion of MAs and the structure findings on OCT B-scan together, for example nearby cystoid spaces in a diabetic eye with macular edema, we might be able to monitor its treatment response. Our study has provided a new prospective to observe flow signal changes inside the MAs, so that we can evaluate the turnover of MAs more objectively.

Couturier et al. [21] analyzed the clinical features of 20 eyes of 14 patients with DR and concluded that FA is more sensitive than OCTA in detecting MAs. They found that only 62% of microaneurysms visualized on FA were detected by OCTA, which is quite similar to the detection rate of MAs on OCTA en face images in our study (112/167, 67.1%). Therefore, OCTA en face image alone is not sensitive enough to observe MAs. MAs observed on OCTA en face images had a broad range of morphologies, including focal bulging, saccular, fusiform, mixed saccular/fusiform, pedunculated, and irregular form [22]. Sometimes it is difficult to differentiate MAs from the capillary background on OCTA en face images, and only MAs with a certain amount of flow signal can be detected on OCTA en face images. The above reasons accounted for the relatively low detection rate of MAs on OCTA.

On OCT enface images, MAs appeared as luminescent ring-shaped morphologies or spots. Our results showed that nearly all the MAs could be located in the corresponding area where angiographically visible MAs lied in. Therefore, if we combine OCT B-scan, OCT en face, and OCTA en face images together, the detection rate of MAs can be raised to about 98.8% in our study.

Our study has some limitations. Firstly, our results showed that flow proportion of MAs might be a possible biomarker to identify leaking MAs, but this is a preliminary result based on small simple size. Larger studies are needed to learn more about this relationship between flow proportion and leakage status of MAs. Secondly, it was a cross-sectional study. The leakage status or flow proportion of MAs was not followed up continuously. This situation deserves special attention in future studies. Thirdly, although potential source of error has been minimized in calculating flow proportion, MA area and flow area were manually selected, so the accuracy of these two components might be lost to some degree. Potential automatic analysis software may help to solve this problem and improve accuracy of flow proportion in the future.

In conclusion, OCT B-scan with flow overlay might be a good alternative to assess leakage of MAs in diabetic eyes. The presence of adjacent cystoid spaces around MAs on OCT B-scan correlated positively with leakage status seen on FA. A combination of OCT B-scan with flow overlay, OCT en face, and OCTA en face imaging appears to be a superior method for detecting MAs when only using OCTA imaging.

## Figures and Tables

**Table 1:** Demographics of patients.

Eyes/patients	39/39
Male/female	20/19
Age, years	58.3 ± 7.2
BCVA (ETDRS letters)	75.3 ± 3.1
Duration of diabetes, years	8.6 ± 4.3
HbA1c, mean ± SD (%)	8.9 ± 2.6
DR stage, *N* eyes (%)	
Mild NPDR	10 (25.6%)
Moderate NPDR	16 (41.1%)
Severe NPDR	13 (33.3%)

DR: diabetic retinopathy; *n*: number.

**Table 2:** Flow proportion of diabetic microaneurysms by OCT B-scan with flow overlay.

Leakage status	*N*	Median	Range
No leakage	36	10.1%	0-53.2%
Mild leakage	52	35.0%	20.2%-86.1%
Severe leakage	79	71.1%	30.3%-95.6%
*P* value		<0.001	

OCT: optical coherence tomography; *N*: number.

**Table 3:** FA leakage status and OCTA/OCT en face visualization of diabetic MAs.

FA leakage status	Number of MAs, *N*	OCTA en face visualization, *N* (%)	OCT en face visualization, *N* (%)
No leakage	36	8 (22.2%)	36 (100%)
Mild leakage	52	35 (67.3%)	52 (100%)
Severe leakage	79	69 (87.3%)	77 (97.5%)

FA: fluorescein angiography; OCTA: optical coherence tomography angiography; OCT: optical coherence tomography; MA: microaneurysm; *N*: number.

## Data Availability

The data used to support the findings of this study are available if needed.
